# Time-on-task decrement in vigilance is modulated by inter-individual vulnerability to homeostatic sleep pressure manipulation

**DOI:** 10.3389/fnbeh.2014.00059

**Published:** 2014-03-06

**Authors:** Micheline Maire, Carolin F. Reichert, Virginie Gabel, Antoine U. Viola, Julia Krebs, Werner Strobel, Hans-Peter Landolt, Valérie Bachmann, Christian Cajochen, Christina Schmidt

**Affiliations:** ^1^Centre for Chronobiology, Psychiatric University Hospital of the University of BaselBasel, Switzerland; ^2^Respiratory Medicine, Department of Internal Medicine, University Hospital BaselBasel, Switzerland; ^3^Chronobiology and Sleep Research, Institute of Pharmacology and Toxicology, University of ZürichZürich, Switzerland; ^4^Clinical Research Priority Program “Sleep & Health”, University of ZürichZürich, Switzerland

**Keywords:** time-on-task, *PER3* polymorphism, sleep deprivation, inter-individual variability, psychomotor vigilance, behavioral vulnerability, sleep loss

## Abstract

Under sleep loss, vigilance is reduced and attentional failures emerge progressively. It becomes difficult to maintain stable performance over time, leading to growing performance variability (i.e., state instability) in an individual and among subjects. Task duration plays a major role in the maintenance of stable vigilance levels, such that the longer the task, the more likely state instability will be observed. Vulnerability to sleep-loss-dependent performance decrements is highly individual and is also modulated by a polymorphism in the human clock gene *PERIOD3 (PER3)*. By combining two different protocols, we manipulated sleep-wake history by once extending wakefulness for 40 h (high sleep pressure condition) and once by imposing a short sleep-wake cycle by alternating 160 min of wakefulness and 80 min naps (low sleep pressure condition) in a within-subject design. We observed that homozygous carriers of the long repeat allele of *PER3 (PER3*^5/5^*)* experienced a greater time-on-task dependent performance decrement (i.e., a steeper increase in the number of lapses) in the Psychomotor Vigilance Task compared to the carriers of the short repeat allele *(PER3*^4/4^*)*. These genotype-dependent effects disappeared under low sleep pressure conditions, and neither motivation, nor perceived effort accounted for these differences. Our data thus suggest that greater sleep-loss related attentional vulnerability based on the *PER3* polymorphism is mirrored by a greater state instability under extended wakefulness in the short compared to the long allele carriers. Our results undermine the importance of time-on-task related aspects when investigating inter-individual differences in sleep loss-induced behavioral vulnerability.

## Introduction

In modern 24/7 society, sleep loss is part of our daily lives, and many professions come along with night or shift work nowadays. The detrimental effects of too little sleep on various domains of cognitive performance have long been known (for a review, see Killgore, 2010). Nevertheless, people are often still able to successfully accomplish complex tasks under such circumstances. Indeed, rather than to lead to a complete loss in the ability to perform, sleep loss induces increasingly greater performance variability (Doran et al., 2001; Durmer and Dinges, 2005; Van Dongen and Dinges, 2005). In other words, optimal performance is still possible even after many hours of sleep deprivation, but at the cost of increasing intermittence of performance lapses e.g., leading to greater standard deviations in reaction times (RT; Doran et al., 2001).

To understand how cognitive performance variation emerges, the two main oscillators involved in the regulation of sleep and wakefulness need to be considered (Borbely, 1982; Daan et al., 1984). On one side, an hourglass-like sleep homeostatic process tracks our sleep-wake history and leads to a rise in sleep propensity or sleep pressure with increasing time awake. On the other side, a circadian process represents a nearly 24-h oscillation, promoting wakefulness and sleep at specific times of the day. The interplay of both processes leads to consolidated states of sleep and wakefulness and contributes to the modulation of cognitive performance over the 24 h light-dark cycle (Cajochen et al., 2004; Dijk and Von Schantz, 2005; Cajochen et al., 2010). Throughout a regular 16-h waking day, cognitive performance remains relatively stable, followed by a steep decrease once wakefulness is extended into the biological night. Most detrimental effects emerge in the early morning hours, when the circadian pacemaker promotes maximal sleep drive and the homeostatic sleep pressure is rather high (after ca. 21–24 h of prior wakefulness) (Wright et al., 2012). With increasing sleep propensity, a certain “wake state instability” (Durmer and Dinges, 2005) is observed, that is, sleep initiating mechanisms tend to progressively interfere with wakefulness. This leads to an increasing performance variability including task disengagement, and a dependency on compensatory mechanisms (Doran et al., 2001; Rogers et al., 2003; Dorrian et al., 2005). To specifically observe this increasing attentional failure, task duration plays a key role—the longer the task, the more likely the growing variability will be detected. This is based on the fact that potential compensatory mechanisms are more likely to fail after a certain time (Doran et al., 2001). Hence, performance variability such as momentary task disengagement does not only depend on prior wakefulness, but also on the duration of the task (Doran et al., 2001).

Vulnerability to performance decrements caused by sleep deprivation and/or adverse circadian phase has been reported to be trait-like (Leproult et al., 2003; Van Dongen et al., 2004, 2005) and to some extent, genetically determined (Landolt, 2008). An increasing body of evidence points toward a variable number tandem repeat (VNTR) polymorphism in the human clock gene *PERIOD3* (*PER3*) to be involved in the modulation of this vulnerability, indicated by a faster build-up and subsequent dissipation of homeostatic sleep pressure in homozygous carriers of the long repeat allele (*PER3*^5/5^carriers) (Viola et al., 2007, 2012; Dijk and Archer, 2009, 2010). In our study, we aimed at investigating the effect of the *PER3* VNTR polymorphism on state instability in vigilance; more precisely, whether the wake-dependent homeostatic increase in the number of performance lapses throughout a 10-min psychomotor vigilance task (PVT) is different in homozygous *PER3* short vs. long allele carriers. The PVT (Dinges and Powell, 1985) has been shown to be sensitive to both sleep deprivation and adverse circadian phase (Wyatt et al., 1999; Graw et al., 2004). With a duration of 10 min, it provides an optimal tool to investigate the time course of vigilance (Doran et al., 2001). We experimentally varied sleep pressure by extending wakefulness to 40 h in one branch of the study (sleep deprivation protocol, SD) and by imposing a short sleep wake-cycle (10 cycles of 160 min of wakefulness and 80 min nap, NP) in the other branch of a balanced cross-over design. We were thus able to investigate momentary attentional failures under systematic homeostatic sleep pressure manipulation over the entire circadian cycle, all in relation to the *PER3* polymorphism. By applying this approach, we previously observed a global increase in the number of lapses during SD compared to NP, and moreover detected a greater number of lapses for *PER3*^5/5^ carriers than *PER3*^4/4^carriers during SD. These results confirmed the adequacy of our protocol to study the trait- and state-like modulation of sleep homeostasis (Maire et al., 2013). However, even though time-on-task decrement has been described to be highly dependent on sleep homeostatic processes and has a significant impact on daily life, the effect of sleep-loss-related trait-like vulnerability has never been reported under this angle. Here, we assumed a generally greater time-on-task effect during SD compared to NP. Further, when compared to the more resilient genotype (*PER3*^4/4^), we expect the more vulnerable genotype (*PER3*^5/5^) to present higher susceptibility to the time-on-task effect when sleep pressure is at high levels, but not when sleep pressure is kept at low levels.

## Materials and methods

### Participants

Twenty-nine healthy volunteers (mean age ± *SD*: 25.38 ± 3.3 years) participated in the study. Table 1 details the demographic data. Fifteen (eight males, seven females) were homozygous carriers of the short repeat allele (*PER3*^4/4^), and 14 (five males, nine females) were homozygous carriers of the long repeat allele (*PER3*^5/5^). The selection of this group was based on the individual's genotype and ability to devote time to participation; the applied exclusion criteria are listed below. All participants completed questionnaires regarding their general and mental health, sleep habits and quality, and chronotype. We excluded participants with general medical, current or past psychiatric and sleep disorders, and usual sleep duration of less than 7 or more than 9 h. Further exclusion criteria encompassed smoking, medication (except oral contraceptives), or drug consumption. To control for circadian phase misalignment, we excluded shift workers, and study applicants who had trans-meridian flights during three months before study participation. A physical examination by the physician in charge as well as a screening night was carried out to exclude sleep disorders, and to habituate the participants to sleep in laboratory conditions with electrodes before study participation. Women who did not use contraceptives (2 out of 16) were tested during the luteal phase of their menstrual cycle. The groups did not significantly differ in terms of sex ratio, age, BMI, bed times preceding study weekends, and questionnaire scores (Table 1). The local ethics committee (Ethikkomission beider Basel, EKBB, Switzerland) approved the study, and all procedures conformed to the standards of the declaration of Helsinki. All participants provided their written informed consent to the participation.

**Table 1:** **Demographic data, questionnaire scores (*M* ± *SD*) and *p*-values derived from *X*^2^- (gender) and *t*-tests (other variables)**.

	***PER3^44^***	***PER3^55^***	***p***
*N* (m, f)	15 (8, 7)	14 (5, 9)	0.34
Age (y)	24.76 (3.38)	25.99 (3.30)	0.22
BMI (kg/m^2^)	21.22 (2.23)	22.62 (2.09)	0.23
Wake time (clock time)	06:49 (56 min)	07:03 (41 min)	0.79
Sleep time (clock time)	22:49 (56 min)	23:03 (41 min)	0.79
PSQI	3.11 (0.99)	2.82 (1.34)	0.66
ESS	3.83 (1.72)	4.09 (1.94)	0.67
MEQ	57.78 (6.94)	55.34 (10.09)	0.22
MCTQ sleep duration (h)	7.93 (0.77)	7.70 (0.60)	0.78
MCTQ MSFsc	4.33 (0.89)	4.02 (1.14)	0.77
MCTQ MSFsac	6.77 (2.90)	6.39 (1.96)	0.73

*PSQI, Pittsburgh Sleep Quality Index (Buysse et al., 1989); ESS, Epworth Sleepiness Scale (Johns, 1991); MEQ, Morningness-Eveningness Questionnaire (Horne and Östberg, 1976); MCTQ, Munich Chronotype Questionnaire (Roenneberg et al., 2003); MSFsc, Mid sleep free days sleep corrected; MSFsac, Mid sleep free days sleep and age corrected*.

### Genotyping

As reported in Maire et al. (2013), DNA was extracted from saliva samples collected with the Oragene DNA sample collection kit using standard procedures (DNA Genotek Inc., Ontario, Canada). All genotypes were determined with an allele-specific PCR with 50 cycles at 60°C. Forward primer: 5′-TTA CAG GCA ACA ATG GCA GT-3′, reverse primer: 5′-CCA CTA CCT GAT GCT GCT GA-3′. Agarose gel (2%) electrophoresis was used to identify the genotype of the individuals.

### Protocol and procedure

**Figure 1:**
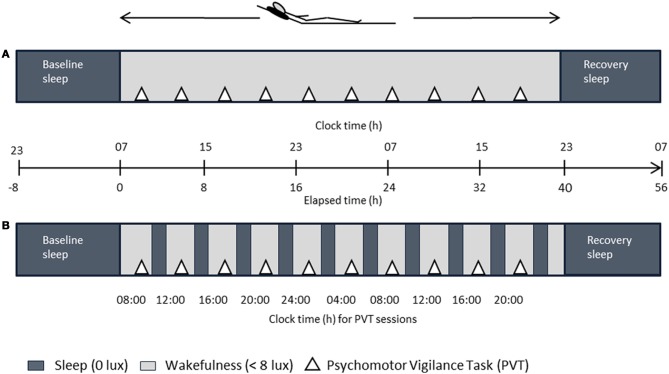
**Overview of the laboratory part**. After baseline sleep (8 h), either a 40-h sleep deprivation **(A)** or a 40-h multiple nap paradigm (**B**, ten 80/160-min sleep/wake cycles) under controlled posture conditions was carried out in a within-subject design, followed by recovery sleep (8 h). Dark gray bars in **(B)** indicate scheduled sleep episodes. Elapsed time indication is relative to 7 a.m. wake up time.

Figure 1 illustrates the study design. Each volunteer completed two study blocks; both comprising an ambulatory part of one week, followed by a 56-h stay in the chronobiology laboratory. During both ambulatory weeks, participants were asked to maintain a regular sleep-wake cycle (8 h ± 30 min time in bed) according to their self-selected sleep-wake timing. Sleep logs and wrist actimetry (Actiwatch®, Cambridge Neurotechnology Ltd., UK) served to control for compliance to the regimen. Participants were requested to abstain from caffeine, alcohol, medication intake (except contraceptive pill), and daytime napping. After each ambulatory part, volunteers reported to the laboratory and underwent the SD and the NP protocol according to a randomized and balanced crossover design. Both protocols started with a baseline night (8 h time in bed at usual bedtimes). After a baseline night, participants stayed awake for 40 h after habitual wake time in the SD; in the NP they underwent 10 alternating cycles of 160 min of scheduled wakefulness (except for the first [120 min] and last wake period [40 min]) and 80 min of scheduled sleep (i.e., naps). Both blocks ended with a recovery night (minimum 8 h time in bed at usual bedtimes) and implied stringently controlled conditions, that are, semi-recumbent posture position in bed during wakefulness, regularly scheduled food intake, dim light (<8 lux) during scheduled wakefulness and zero lux during scheduled sleep episodes (i.e., naps), and no time-of-day indication. Participants' social interaction was restricted to the experimental staff. Getting up was allowed at scheduled times to use the bathroom. During scheduled wakefulness, participants were allowed to read, play card or dice games, and watch selected films. Participants were continuously monitored by electroencephalography (EEG). Data on melatonin, subjective and physiological sleepiness parameters, and polysomnographic nap sleep obtained in this study have been published in Maire et al. (2013).

### Psychomotor vigilance task

Vigilance was assessed by a modified version of the PVT (Dinges and Powell, 1985) at ten time points within a test session of approximately 30 min duration, also encompassing an unrelated working memory test. The first session started after 1h awake and testing was subsequently repeated every 4 h until the end of the protocol (clock times see Figure 1). The PVT was the second test in each session and started at about 20 min into the test bout, after the working memory task. Every second cognitive test session took place in a functional magnetic resonance imaging (fMRI) scanner. In the PVT, a fixation cross was presented on a black screen. At random intervals (2–10 sec), a millisecond counter started, and participants were instructed to press a button to stop the counter as fast as possible (clock event). Modification of the original task consisted in the inclusion of null events, where the clock event was replaced by the fixation cross (25% of the trials at random) due to fMRI experimental design compatibility. Feedback of RT performance was displayed for one sec after the participants' response. Altogether, the task duration was 10 min. Here we report lapses (RT > 500 ms), optimal performance (the fastest 10% of the RTs between 150 and 500 ms, to exclude anticipatory responses and lapses, respectively), and standard deviations of the RTs. According to Basner and Dinges (2011), lapses represent the most sensitive measure to investigate the effects of acute total sleep deprivation, whereas the fastest RTs often remain unaffected by SD (Graw et al., 2004). Standard deviations of RTs were analyzed as a marker of performance variability within subjects (Doran et al., 2001).

### Effort scales

After every test session, visual analog scales (VAS) ranging from 0–100 were used to assess subjectively perceived effort during the task, ranging from *“little”* to *“much.”* Participants had to indicate experienced *strain, concentration, fatigue*, and *motivation* during the test.

### Statistical analysis

All analyses were performed using the statistical package SAS (SAS Institute Inc., Cary, NC; version 9.3). Variables were analyzed with mixed-model repeated measures ANOVAs (PROC MIXED) and *p*-values were based on Kenward-Roger's corrected degrees of freedom (Kenward and Roger, 1997). Alpha was set at 0.05. Contrasts were assessed using the LSMEANS statement. For *post-hoc* analysis, the Tukey-Kramer test was applied for alpha-adjustments of multiple comparisons, and corrected *p*-values are reported. For global PVT analysis (lapses and 10% fastest RTs), the factors *genotype* (*PER3*^5/5^ vs. *PER3*^4/4^), *condition* (NP vs. SD), and *time* (10 sessions) were used. *Time* represents time elapsed into the protocol starting at habitual wake time (see Table 1 for average wake times per genotype). For the time-on-task analysis, we included the factors *genotype, time*, and *time-on-task* (first three minutes vs. last three minutes), and analyzed each condition separately for lapses, fastest RTs, and standard deviations. For graphs, 7 a.m. was used as the average reference wake up time. The lapses were transformed (transformation by $\sqrt{x}$ + $\sqrt{x}$ + 1; for details, see Graw et al. (2001), and subsequently *z*-transformed due to different testing environment (every second session took place in the fMRI scanner with a different response keypad). Fastest RTs and standard deviations were equally *z*-transformed to account for the reason stated above. The first two trials of each test were excluded from analysis to eliminate effects of orienting to the task.

## Results

### Global PVT performance

#### Lapses

**Figure 2:**
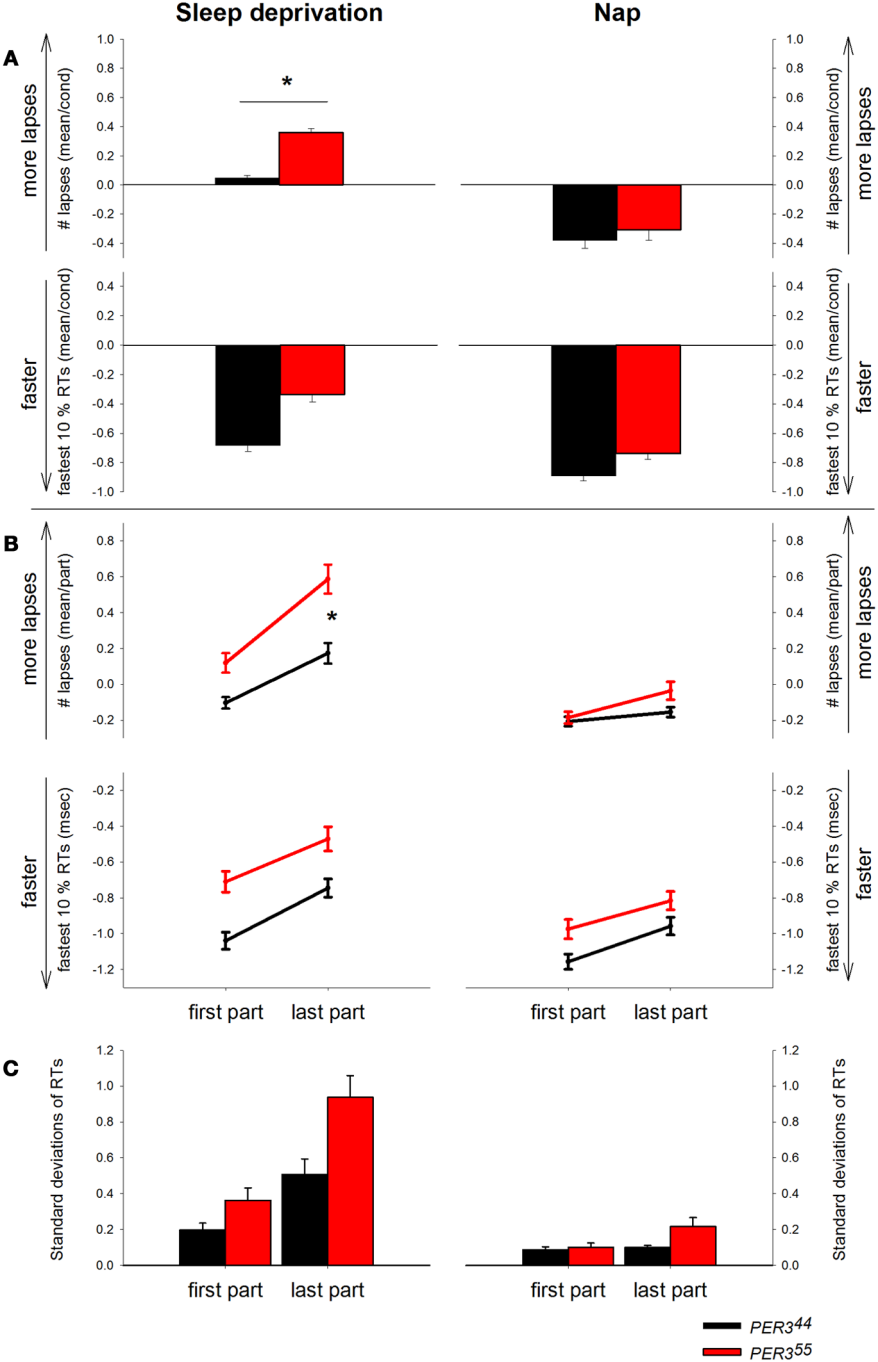
**Psychomotor Vigilance Task (PVT) performance displayed by genotype and condition**. *RT* = Reaction time. **(A)** Mean number of lapses (transformed) and mean of the 10% fastest *RT*s (*z*-scores) during sleep deprivation and during the nap protocol by genotype (*PER3*^5/5^: red bars, *PER3*^4/4^: black bars). Asterisk represents *p*-value < 0.05. **(B)** PVT lapses and fastest RTs displayed by genotype and condition over the first and the last part of the task over all sessions. *PER3*^5/5^: red lines, *PER3*^4/4^: black lines. **(C)** Standard deviations of RTs plotted by genotype and condition for the first and the last test part. *PER3*^5/5^: red bars, *PER3*^4/4^: black bars.

*PER3*^5/5^ carriers produced significantly more lapses than *PER3*^4/4^ carriers in the SD (interaction: *condition x genotype* [*F*_(1, 513)_ = 18.17, *p* < 0.0001]; see Figure 2A (*PER3*^5/5^; 0.62 ± 0.10, vs. *PER3*^4/4^; 0.08 ± 0.08; mean ± SE; *p* = 0.0323), while during the NP protocol, no significant difference between the two genotypes was observed (see also Maire et al., 2013).

#### Optimal performance

Although lapses in performance increase under SD, normal RTs are still possible (Doran et al., 2001). Therefore, we were interested in the 10% fastest RTs representing the optimal performance levels in the respective task session. Analyses (Figure 2A) revealed main effects of *condition* [*F*_(1, 512)_ = 23.27, *p* ≤ 0.0001] and *time* [*F*_(1, 512)_ = 9.94, *p* ≤ 0.0001], with faster optimal RTs during NP (−0.14 ± 0.05, mean ± SE) than SD (0.14 ± 0.06, mean ± SE), and during the biological day compared to night time. The interaction *time × condition* was significant [*F*_(1, 512)_ = 2.42, *p* = 0.011], indicating that during the last session of the SD protocol (8 p.m., 37 h awake), participants had significantly higher (slower) optimal RTs (*p* = 0.0031) than during NP. There was no main effect of *genotype* or significant interactions were revealed regarding this factor (*p*_*all*_ > 0.05).

### Time-on-task effects

#### Lapses

**Figure 3:**
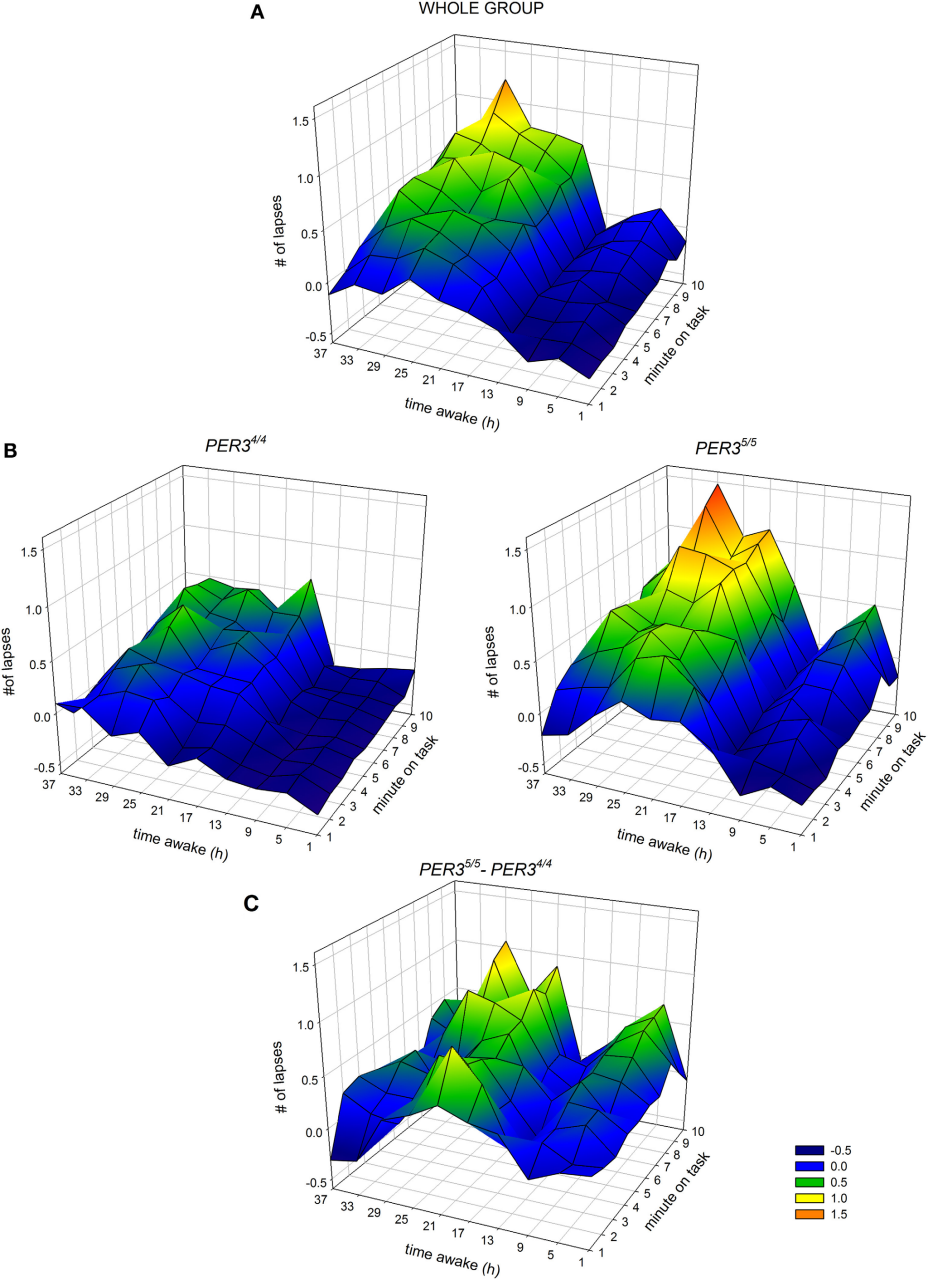
**Interaction between hours of scheduled wakefulness** (time awake) **during sleep deprivation** (y-axis of each panel) **and time-on-task** (minutes on task, *x*-axis of each panel) **in the modulation of the number of lapses on the Psychomotor Vigilance Task** (PVT, *z*-axis of each panel) **(A)** for the whole group, **(B)** for *PER3*^4/4^ carriers (left) and *PER3*^5/5^ carriers (right) and **(C)** the difference between the two genotypes (*PER3*^5/5^–*PER3*^4/4^). Higher values on the *z*-axis indicate higher levels of impairment.

The time course of the lapses during SD over the 10-min task duration and for all sessions is shown in Figure 3. **(A)** depicts the whole group; **(B)** shows each genotype separately and **(C)** illustrates the difference between genotypes. The analysis yielded a significant main effect of *genotype* with *PER3*^5/5^ carriers showing overall more lapses during SD (Table 2; *PER3*^5/5^: 0.35 ± 0.05 vs. *PER3*^4/4^: 0.03 ± 0.03; mean ± SE), confirming the global PVT performance results. Both factors, *time* and *time-on-task*, were significant, showing that lapses varied with test timing and were more numerous during the last portion of the 10-min PVT task (Table 2; first section: −0.009 ± 0.03; last section: 0.37 ± 0.05; mean ± SE). Also, the interaction *time × time-on-task* was significant (Table 2), such that during the night session (clock time: 4 a.m.) as well as during two sessions at noon and in the afternoon of the second day during the SD (clock times: 12 p.m. and 4 p.m.), the lapses during the last test part were more numerous (*p*_*all*_ < 0.05). Interestingly, the effect of *time* was modulated by *genotype* (Table 2), indicating that *PER3*^5/5^genotypes produced significantly more lapses in the session during the night compared to *PER3*^4/4^ carriers (21 h awake, clock time 4 a.m., *p* < 0.05; *PER3*^5/5^; 0.36 ± 0.07, vs. *PER3*^4/4^; 0.13 ± 0.05; mean ± SE). Likewise, a significant interaction *time-on-task × genotype* (Table 2) revealed that while both groups showed a time-on-task-dependent increase in lapses, *PER3*^5/5^ carriers had significantly more lapses during the last test section when compared to *PER3*^4/4^carriers (Figure 2B; *p* < 0.01; *PER3*^5/5^; 0.59 ± 0.08, vs. *PER3*^4/4^; 0.17 ± 0.06; mean ± SE), whereas both groups did not differ in the first test section (*p* > 0.1).

**Table 2:** **Results of mixed model ANOVA for time-on-task effects; *F*-values (df), and *p*-values**.

		***PER3***	**T**	**ToT**	**T × ToT**	***PER3* × T**	**ToT × *PER3***	**T × ToT × *PER3***
***SD***	Lapses	*F*_(1, 27)_ = 5.59	*F*_(9, 511)_ = 20.97	*F*_(1, 511)_ = 80.99	*F*_(9, 511)_ = 2.14	*F*_(9, 511)_ = 2.95	*F*_(1, 511)_ = 5.6	*F*_(9, 511)_ = 1.47
		***p* = 0.0255**	***p* < 0.0001**	***p* < 0.0001**	***p* = 0.0248**	***p* = 0.002**	***p* = 0.0184**	*p* = 0.16
	Fast RT	*F*_(1, 27)_ = 3.36	*F*_(9, 507)_ = 16.31	*F*_(1, 507)_ = 44.55	*F*_(9, 507)_ = 0.21	*F*_(9, 507)_ = 0.54	*F*_(1, 507)_ = 0.53	*F*_(1, 507)_ = 1.12
		*p* = 0.07	***p* < 0.0001**	***p* < 0.0001**	*p* = 0.99	*p* = 0.85	*p* = 0.47	*p* = 0.35
***NP***	Lapses	*F*_(1, 27)_ = 0.78	*F*_(9, 513)_ = 12.86	*F*_(1, 513)_ = 10.66	*F*_(9, 513)_ = 2.54	*F*_(9, 513)_ = 2.13	*F*_(1, 513)_ = 3.01	*F*_(9, 513)_ = 0.32
		*p* = 0.3842	***p* < 0.0001**	***p* = 0.0012**	***p* = 0.0096**	***p* = 0.0259**	*p* = 0.08	*p* = 0.97
	Fast RT	*F*_(1, 27)_ = 1.04	*F*_(9, 512)_ = 16.32	*F*_(1, 512)_ = 35.08	*F*_(9, 512)_ = 1.91	*F*_(9, 512)_ = 1.76	*F*_(1, 512)_ = 0.22	*F*_(9, 512)_ = 0.65
		*p* = 0.31	***p* < 0.0001**	***p* < 0.0001**	***p* = 0.048**	*p* = 0.07	*p* = 0.64	*p* = 0.75

*Significant results (p < 0.05) are printed in bold for factors genotype (PER3), time, T; time on task, ToT; and interactions*.

Under low sleep pressure (NP), significant *time* and *time-on-task* effects revealed a time-of-day-dependent pattern and an increase in lapses over the course of the task (Table 2; first section: − 0.18 ± 0.02; last section: − 0.09 ± 0.03; mean ± SE, Figure 2B). The interaction *time x time-on-task* (Table 2) showed that especially in session 7 (8 a.m. on the second day of the protocol), lapses increased from the first to the last section (*p* = 0.0006). However, opposed to what was seen during SD, we observed no significant main effect of *genotype* or *genotype × time-on-task* (Table 2).

#### Optimal performance

The analysis of the 10% fastest RTs (Figure 2B) during SD revealed a significant effect of *time* and *time-on-task* (Table 2), indicating, as expected, that RTs were lower during the biological day and within the first part of the test (First part: − 0.88 ± 0.03 vs. last part: − 0.61 ± 0.04, mean ± SE). No significant interaction was found for *time × time-on-task* (Table 2). Although there was a trend for a main effect of *genotype*, no significant interactions were revealed regarding this factor (Table 2, Figure 2B).

During NP, we observed a significant effect of *time* and *time-on-task*, equally showing faster RTs during the biological day and the first test part (Table 2). Here, the interaction *time × time-on-task* was significant, indicating that during the tests at 8 a.m. on both days, RTs were significantly lower in the first test part (Table 2). *Genotype* and the interactions with this factor were not significant (Table 2, Figure 2B).

### Standard deviations of reaction times

The analysis of the standard deviations of RTs (Figure 2C) throughout the task during SD revealed significant main effects of *time* [*F*_(9, 511)_ = 14.54, *p* ≤ 0.0001] and *time-on-task* [*F*_(1, 511)_ = 41.21, *p* ≤ 0.0001], as well as the interaction of these two factors [*F*_(9, 511)_ = 3.39, *p* = 0.0005]. In other words, the standard deviations were increasing with time awake, to reach a maximum at noon on the second sleep deprived day (12 p.m.), and decreased again toward the biological evening. This pattern was more pronounced in the last part of the test. Although showing a trend, the main effect of *genotype* was not significant [*F*_(1, 27)_ = 3.28, *p* = 0.0812]. The interaction of *time × genotype* was significant [*F*_(9, 511)_ = 2.9, *p* = 0.0024], as well as the interaction of *time-on-task × genotype* [*F*_(9, 511)_ = 3.89, *p* = 0.0491]. Post hoc tests revealed that the standard deviations of the RTs differed between the genotypes mainly in the noon-session of the second day (12 p.m., *p* < 0.0001). Moreover, the genotypes did not differ did in terms of their variability of RTs in first test part (*p* = 0.36), but showed a trend for a difference in standard deviations during the last test part (*p* = 0.0734). The three-way interaction between all factors was not significant (*p* > 0.1).

In the NP, the main effects of *time* [*F*_(9, 513)_ = 8.25, *p* < 0.0001] and *time-on-task* [*F*_(1, 513)_ = 6.09 *p* = 0.0139] were significant, as was the interaction of these two factors [*F*_(9, 513)_ = 2.12, *p* = 0.0263], showing an increase of the standard deviations toward the biological morning, which was more pronounced in the last test minutes. The effect of the factor *genotype* was not significant [*F*_(1, 27)_ = 1.68, *p* = 0.2059]. However, the interaction of *genotype* with *time* [*F*_(9, 513)_ = 3.97, *p* < 0.0001] was significant, showing greater standard deviations in *PER3*^5/5^carriers during the session at 8 a.m. on the second day compared to the short allele carriers (*p* < 0.0001). Although a significant interaction of *genotype* × *time-on-task* [*F*_(1, 513)_ = 4.1, *p* = 0.0435] was revealed, none of the post hoc comparisons showed significant differences between genotypes.

### Effort scales

None of the items on the VAS questionnaire regarding perceived strain, extent of concentration, fatigue, or motivation of participants during task performance differed significantly between genotypes (*p_all_* > 0.05; data not shown). Neither did the genotypes differ significantly in terms of these indicators across time (*genotype × time, p* > 0.05). However, we observed significant main effects of *time* (*p_all_* < 0.05) for all four variables, indicating a time-of-day-dependent variation for the whole group. Significant main effects of *condition* for strain, concentration, and fatigue (*p_all_* < 0.0001) revealed higher values during SD, whereas motivation for the task was comparable during both conditions (*p* > 0.05).

## Discussion

As hypothesized, *PER3*^5/5^ carriers had significantly more difficulties to maintain stable attentional performance over a period of 10 min than *PER3*^4/4^carriers, particularly under conditions of high sleep pressure and at times when the circadian pacemaker promotes sleep. When sleep pressure was kept at low levels by multiple naps, the groups performed equally and no genotype-modulated pattern of a time-on-task decrement was observed. Momentary task disengagement seems to be more pronounced in *PER3*^5/5^than in *PER3*^4/4^ carriers under SD—thus, they suffered more from elevated sleep pressure conditions. Importantly, no genotype-related difference in subjectively perceived strain, effort or motivation was found in either of the protocols. By analysing the 10% fastest RTs (i.e., optimal performance), we showed that the time course of optimal performance levels did not differ in function of genotype, indicating that a temporary mobilization of effort is still possible for both vulnerable and more resilient participants. The differential extent of the resulting variability in RTs is mirrored in the standard deviations being greater for *PER3*^5/5^carriers. A faster homeostatic build-up of sleep pressure in *PER3*^5/5^carriers than in *PER3*^4/4^carriers has been reported (Viola et al., 2007, 2012; Goel et al., 2009), as indexed by more slow wave sleep and more EEG slow-wave activity in *PER3*^5/5^carriers. Moreover, the deterioration in cognitive performance, operationalized as a composite of several cognitive tasks (Viola et al., 2007) as well as working memory (Groeger et al., 2008), was shown to be greater in *PER3*^5/5^compared to *PER3*^4/4^carriers under SD, which was paralleled by an increase in physiological correlates of sleepiness, such as EEG theta activity and the incidence of slow eye movements (SEM) (Viola et al., 2007; Groeger et al., 2008). Likewise, we have previously reported that our *PER3*^5/5^sample produced a greater number of PVT lapses, and higher values on subjective and physiological indicators of sleepiness under high sleep pressure conditions (Maire et al., 2013). However, other authors could not find differences in PVT performance between the genotypes (Goel et al., 2009; Kuna et al., 2012 [lapses]; Lo et al., 2012 [lapses and inverse of the 10% slowest RT]). This discrepancy could be related to the fact that in contrast to others, we strictly controlled for the amount of prior wakefulness and circadian phase by systematically manipulating these two processes in a SD and a nap protocol which allowed for an accurate titration and quantification of the circadian and homeostatic influence on attentional failures over a rather long time span (40 h).

Task duration is an important feature of the demand level a cognitive task exerts. The interplay between sleep deprivation, state instability and task duration has been described before (for an overview, see Doran et al., 2001). However, this phenomenon is rarely reported when studying the impact of sleep loss on cognition, and has not yet been investigated with respect to inter-individual differences in the behavioral vulnerability to sleep loss. Early theories associated vigilance decrement over a certain time span on the task with the monotonous and repetitious nature of vigilance tasks (for a review, see Warm et al., 2008). More recent studies show that the maintenance of stable vigilance levels also depends on task type and its workload, and that the temporal irregularity of the stimuli contributes majorly to the level of demands such a task has (Warm et al., 2008). Zhou et al. (2011) recently showed that performance variability is greater the longer one has been awake prior to performance and the closer to the circadian nadir (i.e., early morning hours). Although the variability detected in their study was not related to the duration of the task *per se*, the authors suggest that state instability acts as an explanation for the responsiveness of neurobehavioral performance to increasing sleep drive already during a habitual wake period.

Importantly, Doran et al. (2001) state that lapses will progress into uncontrolled sleep attacks due to increasing homeostatic sleep pressure. In line with this, we have recently shown that *PER3*^5/5^carriers indeed have more incidental SEM as well as unintentional sleep attacks during SD, particularly during the biological night (Maire et al., 2013). Thus, with our findings of *PER3*^5/5^ carriers showing a greater increase in attentional lapses, we confirm that the responsiveness to SD is greater in this group and that their stronger sleep homeostatic process might be mirrored in the time course of performance. Interestingly, genotypes did not differ in terms of their optimal RTs, although we observed a general time-on-task effect for this measure, too. Indeed, optimal RTs in the PVT seem to be only marginally affected by elevated sleep pressure during a 40-h SD protocol (Graw et al., 2004).

Several studies (Drummond et al., 2005; Weissman et al., 2006; Chee et al., 2008) have linked lapses in performance to a lower deactivation of the so-called brain default mode network initially presented in Raichle et al. (2001). Furthermore, a recent study by Asplund and Chee (2013) showed that both sleep deprivation and time-on-task lead to reduced activation in overlapping brain areas, suggesting that these effects have shared neural and psychological causes. An fMRI study by Vandewalle et al. (2009) showed differences in activations for *PER3*^5/5^carriers compared to *PER3*^4/4^ carriers after 25 h of SD during a working memory task. Specifically, *PER3*^4/4^ carriers showed no reductions in activations, but were able to recruit supplemental brain areas, while *PER3*^5/5^carriers showed widespread reductions in brain activations after SD. The recruitment of supplemental brain areas might mirror compensatory effects (Drummond et al., 2000) that are necessary to prevail against task disengagement. It remains to be determined how the greater vulnerability of *PER3*^5/5^carriers to time-on-task-dependent attentional failures is mirrored at the cerebral level, and whether brain activation differs where optimal performance can be sustained under sleep loss.

Motivation plays a major role in successfully performing a task, and might even mask the more serious effects of sleep deprivation through compensatory effort (Doran et al., 2001). Indeed, the mobilization of effort to keep attentional performance stable despite challenging sleep loss conditions seems to depend largely on motivation (see Sarter et al., 2006 for a review). According to our data, subjectively perceived motivation was comparable between genotypes, also indicated by the fact that “normal” RTs still occurred (Doran et al., 2001). Thus, we conclude that the difference we observed results mainly from divergent sleep homeostatic forces acting on wake state instability, as it is obviously not obscured by discrepancies in motivation. Besides the sleep homeostatic forces only, the interplay between homeostatic and circadian sleep promotion in the early morning could also be altered in the more vulnerable genotype (i.e., *PER3*^5/5^), since most of the differences in attentional failures between the two groups occurred after 21–25 h of extended wakefulness, which corresponded to the circadian sleep maintenance zone between 4 and 8 a.m. in our subject sample. Presumably, these differences in sleep homeostatic and/or circadian drives might allow or hinder the activation of attentional top-down mechanisms at the cerebral level. A possible explanation could be increased prefrontal cortex (PFC) cholinergic activity that might activate the anterior attention system, favoring the top-down optimization of input processing in sensory regions (Sarter et al., 2006). Hence, cholinergic PFC control may optimize goal-directed behavior and cognitive processes, despite performance challenges, such as time-on-task, circadian phase shifts, and sleep loss (Sarter et al., 2006).

Taken together, we show that attentional performance lapses in the PVT reflect the failure to stay focused on the task—which was significantly more difficult for *PER3*^5/5^than *PER3*^4/4^ carriers. However, optimal performance and thus temporary mobilization of effort throughout the task did not depend on genotype. A probable limitation of our study is the rather small sample size. However, by carefully selecting young, healthy participants without sleep complaints and controlling for gender ratio, chronotype, sleep duration, and timing across groups, we chose a rather homogenous phenotype to maximize potential contribution of the *PER3* polymorphism to vulnerability in combination with highly controlled laboratory conditions that restrict potential masking factors such as light influence, body posture, or social and nutritional timing cues.

This is the first study to report time-on-task effects modulated by the *PER3* polymorphism by combining two protocols with low and high sleep pressure levels. Our results provide further evidence that the *PER3* polymorphism is implicated in inter-individual differences in the susceptibility to sleep loss. As momentary lapses in attention can have severe consequences in professional and daily live, our results undermine the importance of considering the time course of performance in further investigations of the nature of sleep loss-related inter-individual differences in cognitive performance.

### Conflict of interest statement

The authors declare that the research was conducted in the absence of any commercial or financial relationships that could be construed as a potential conflict of interest.
